# Crystal structure of 3-chloro-*N*-(2-nitro­phen­yl)benzamide

**DOI:** 10.1107/S2056989015014620

**Published:** 2015-08-22

**Authors:** Rodolfo Moreno-Fuquen, Alexis Azcárate, Alan R. Kennedy

**Affiliations:** a Departamento de Química, Facultad de Ciencias Naturales y Exactas, Universidad del Valle, Apartado 25360, Santiago de Cali, Colombia; b WestCHEM, Department of Pure and Applied Chemistry, University of Strathclyde, 295 Cathedral Street, Glasgow G1 1XL, Scotland

**Keywords:** crystal structure, benzamide, hydrogen bonding, halogen–halogen inter­actions

## Abstract

In the title compound, C_13_H_9_ClN_2_O_3_, the mean plane of the central amide fragment (r.m.s. deviation = 0.016 Å) subtends dihedral angles of 15.2 (2) and 8.2 (2)° with the chloro- and nitro-substituted benzene rings, respectively. An intra­molecular N—H⋯O hydrogen bond generates an *S*(6) ring. In the crystal, mol­ecules are linked by weak C—H⋯O hydrogen bonds, forming *C*(7) chains which propagate along [010], but no Cl⋯Cl short contacts are observed.

## Related literature   

For halogen–halogen inter­actions in benzanilide compounds, see: Vener *et al.* (2013 ▸); Nayak *et al.* (2011 ▸).
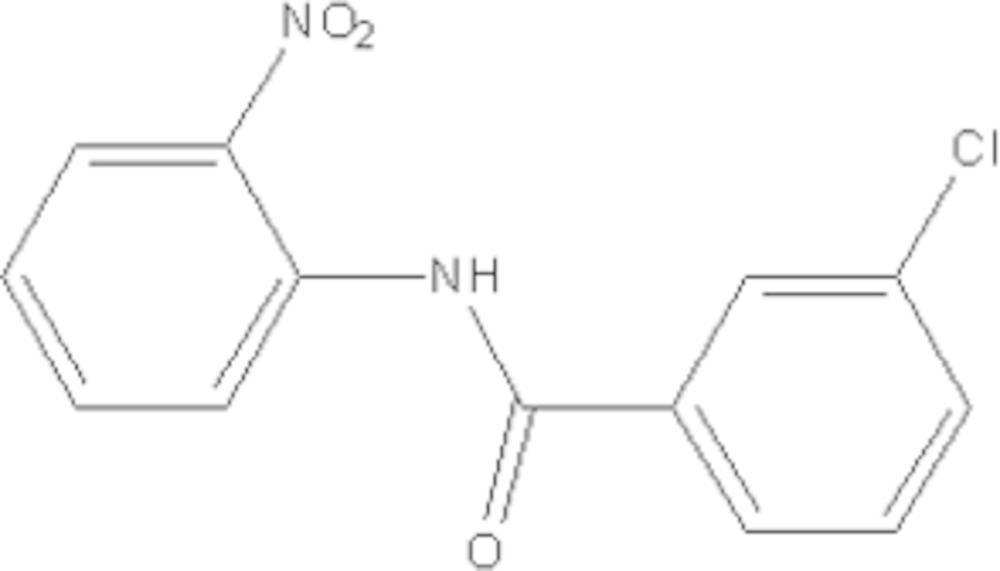


## Experimental   

### Crystal data  

C_13_H_9_ClN_2_O_3_
*M* *_r_* = 276.67
Monoclinic, 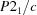

*a* = 12.6300 (9) Å
*b* = 14.1462 (12) Å
*c* = 6.7797 (6) Å
β = 105.475 (7)°
*V* = 1167.39 (17) Å^3^
*Z* = 4
Mo *K*α radiation
μ = 0.33 mm^−1^
*T* = 123 K
0.40 × 0.08 × 0.05 mm

### Data collection  

Oxford Diffraction Gemini S diffractometer
Absorption correction: multi-scan (*CrysAlis PRO*; Oxford Diffraction, 2010 ▸) *T*_min_ = 0.839, *T*_max_ = 1.000
10366 measured reflections
10366 independent reflections
7015 reflections with *I* > 2σ(*I*)

### Refinement  

*R*[*F* ^2^ > 2σ(*F* ^2^)] = 0.068
*wR*(*F* ^2^) = 0.179
*S* = 1.00
10367 reflections
177 parameters
H atoms treated by a mixture of independent and constrained refinement
Δρ_max_ = 0.78 e Å^−3^
Δρ_min_ = −0.49 e Å^−3^

### 

Data collection: *CrysAlis PRO* (Oxford Diffraction, 2010 ▸); cell refinement: *CrysAlis PRO*; data reduction: *CrysAlis PRO*; program(s) used to solve structure: *SIR92* (Altomare *et al.*, 1994 ▸); program(s) used to refine structure: *SHELXL2014* (Sheldrick, 2015 ▸); molecular graphics: *ORTEP-3 for Windows* (Farrugia, 2012 ▸) and *Mercury* (Macrae *et al.*, 2006 ▸); software used to prepare material for publication: *WinGX* (Farrugia, 2012 ▸).

## Supplementary Material

Crystal structure: contains datablock(s) I, global. DOI: 10.1107/S2056989015014620/hb7476sup1.cif

Structure factors: contains datablock(s) I. DOI: 10.1107/S2056989015014620/hb7476Isup2.hkl

Supporting information file. DOI: 10.1107/S2056989015014620/hb7476Isup3.cml

**Fig. 1.**
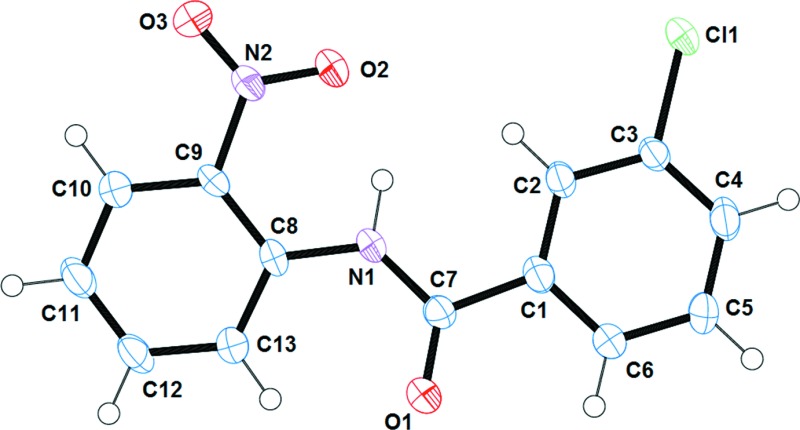
The molecular structure of (I) with displacement ellipsoids drawn at the 50% probability level. H atoms are shown as spheres of arbitrary radius.

. DOI: 10.1107/S2056989015014620/hb7476fig1.tif
The mol­ecular structure of (I) with displacement ellipsoids drawn at the 50% probability level. H atoms are shown as spheres of arbitrary radius.

**Fig. 2.**
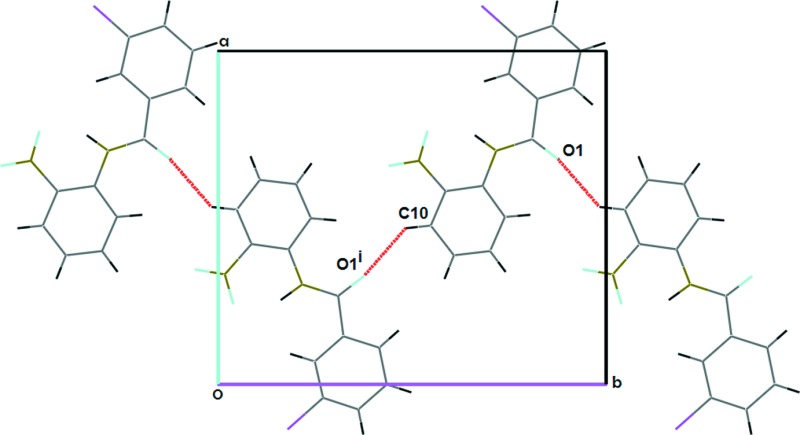
Part of the crystal structure of (I), showing the formation of C(7) chains along [010] [Symmetry code: (i) -x + 1, y - 1/2, -z + 3/2].

Click here for additional data file.x y z . DOI: 10.1107/S2056989015014620/hb7476fig2.tif
Part of the crystal structure of (I), showing the formation of C(7) chains along [010] [Symmetry code: (i) −*x* + 1, *y* − 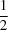, −*z* + 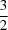].

CCDC reference: 1416793

Additional supporting information:  crystallographic information; 3D view; checkCIF report

## Figures and Tables

**Table 1:** Hydrogen-bond geometry (, )

*D*H*A*	*D*H	H*A*	*D* *A*	*D*H*A*
N1H1*N*O2	0.98(7)	1.75(7)	2.612(6)	144(6)
C10H10O1^i^	0.95	2.39	3.158(7)	138

Symmetry code: (i) 
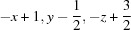
.

## supplementary crystallographic information

### S1. Comment

The crystal structure determination of 3-chloro-N-(2-nitrophenyl)benzamide (I), is part of a study on benzanilides carried out in our research group, and it was obtained from the reaction between 3-chlorobenzoic acid and 2-nitroaniline mediated by the presence of thionyl chloride. The study of intermolecular halogen-halogen interactions is a current problem and several authors have presented interesting results. Halogen-halogen short interactions, in other similar studies, show Cl···Cl distances of the order of 3.8 Å. Theoretical studies of density analysis, varying the Cl···Cl distance from 3.0 to 4.0 Å, using DFT solid state program, have been undertaken (Vener *et al.*, 2013). Geometric considerations in halogen-halogen interactions, for various benzanilide systems, showed different behaviors. Interactions of fluorine with other halogens Cl, Br, I, in different benzanilide systems, include interactions type: trans, cis or L-geometry (Nayak *et al.*, 2011). The molecular structure of (I) is shown in Fig. 1. The central amide moiety, C8—N1-C7(═O1)—C1, is essentially planar (r.m.s. deviation for all non-H atoms = 0.0164 Å) and it forms dihedral angles of 15.2 (2)° with the C1-C6 and 8.2 (2)° with the C8-C13 rings respectively. In the crystal structure (Fig. 2), molecules are linked by weak C-H···O intermolecular contacts. The C10-H10···O1 hydrogen bond interactions are responsible for crystal growth parallel to (2 0 -2). In this interaction, the C-H in the molecule at (x,y,z) acts as a hydrogen-bond donor to O1 atom of the carbonyl group at (-x+1,+y-1/2,-z+3/2). These interactions generate C(7) chains of molecules along [010]. Other intra N-H···O and N-H···N are observed (see Table 1, Nardelli, 1995). The shorest Cl···Cl contact distance in this structure is 3.943 (3) Å.

### S2. Experimental

The title molecule was synthesized taking 0.200 g (1.270 mmol) of 3-chlorobenzoic acid and it was placed under reflux with 2 mL of thionyl chloride for two hours. After this time an equimolar amount of o-nitroaniline, dissolved in 10 mL of acetonitrile and allowed to reflux at constant stirring for 3 hours was added. The final solution was left to slow evaporation to obtain yellow crystals. [m.p. 399 (1)K].

### S3. Refinement

All Hm atoms were positioned in geometrically idealized positions, C—H = 0.95 Å, and were refined using a riding-model approximation with *U*_iso_(H) constrained to 1.2 times *U*_eq_ of the respective parent atom. H1N atom was found from the Fourier maps and its coordinates were refined freely.

### Crystal data

**Table:**

C_13_H_9_ClN_2_O_3_	*D*_x_ = 1.574 Mg m^−^^3^
*M**_r_* = 276.67	Melting point: 399(1) K
Monoclinic, *P*2_1_/*c*	Mo *K*α radiation, λ = 0.71073 Å
*a* = 12.6300 (9) Å	Cell parameters from 10366 reflections
*b* = 14.1462 (12) Å	θ = 3.3–27.0°
*c* = 6.7797 (6) Å	µ = 0.33 mm^−^^1^
β = 105.475 (7)°	*T* = 123 K
*V* = 1167.39 (17) Å^3^	Needle, yellow
*Z* = 4	0.40 × 0.08 × 0.05 mm
*F*(000) = 568	

### Data collection

**Table:**

Oxford Diffraction Gemini S diffractometer	10366 independent reflections
Radiation source: fine-focus sealed tube	7015 reflections with *I* > 2σ(*I*)
Graphite monochromator	*R*_int_ = 0.000
ω scans	θ_max_ = 29.0°, θ_min_ = 3.3°
Absorption correction: multi-scan (*CrysAlis PRO*; Oxford Diffraction, 2010)	*h* = −17→17
*T*_min_ = 0.839, *T*_max_ = 1.000	*k* = −17→17
10366 measured reflections	*l* = −9→8

### Refinement

**Table:**

Refinement on *F*^2^	Primary atom site location: structure-invariant direct methods
Least-squares matrix: full	Secondary atom site location: difference Fourier map
*R*[*F*^2^ > 2σ(*F*^2^)] = 0.068	Hydrogen site location: inferred from neighbouring sites
*wR*(*F*^2^) = 0.179	H atoms treated by a mixture of independent and constrained refinement
*S* = 1.00	*w* = 1/[σ^2^(*F*_o_^2^) + (0.0657*P*)^2^] where *P* = (*F*_o_^2^ + 2*F*_c_^2^)/3
10367 reflections	(Δ/σ)_max_ < 0.001
177 parameters	Δρ_max_ = 0.78 e Å^−^^3^
0 restraints	Δρ_min_ = −0.49 e Å^−^^3^

### Special details

**Table:**

Experimental. IR spectra was recorded on a FT—IR SHIMADZU IR-Affinity-1 spectrophotometer. IR (KBr), cm^-1^, 3348 (amide N–H); 1684 (amide, C=O); 1499 and 1342 (-NO_2_)Absorption correction: CrysAlisPro, Agilent Technologies, Version 1.171.34.46 (release 25-11-2010 CrysAlis171 .NET) (compiled Nov 25 2010,17:55:46) Empirical absorption correction using spherical harmonics, implemented in SCALE3 ABSPACK scaling algorithm.
Geometry. All esds (except the esd in the dihedral angle between two l.s. planes) are estimated using the full covariance matrix. The cell esds are taken into account individually in the estimation of esds in distances, angles and torsion angles; correlations between esds in cell parameters are only used when they are defined by crystal symmetry. An approximate (isotropic) treatment of cell esds is used for estimating esds involving l.s. planes.
Refinement. Refinement of *F*^2^ against ALL reflections. The weighted *R*-factor *wR* and goodness of fit *S* are based on *F*^2^, conventional *R*-factors *R* are based on *F*, with *F* set to zero for negative *F*^2^. The threshold expression of *F*^2^ > σ(*F*^2^) is used only for calculating *R*-factors(gt) *etc*. and is not relevant to the choice of reflections for refinement. *R*-factors based on *F*^2^ are statistically about twice as large as those based on *F*, and *R*- factors based on ALL data will be even larger.

### Fractional atomic coordinates and isotropic or equivalent isotropic displacement parameters (Å^2^)

**Table:**

	*x*	*y*	*z*	*U*_iso_*/*U*_eq_	
Cl1	−0.13525 (11)	0.17880 (10)	0.1877 (3)	0.0345 (4)	
O1	0.3249 (3)	0.3758 (3)	0.5550 (7)	0.0413 (11)	
O2	0.2403 (3)	0.0390 (3)	0.4902 (6)	0.0417 (12)	
O3	0.3446 (4)	−0.0604 (3)	0.6933 (7)	0.0467 (13)	
N1	0.2996 (4)	0.2164 (3)	0.5084 (7)	0.0270 (11)	
N2	0.3297 (4)	0.0170 (4)	0.6117 (7)	0.0306 (12)	
C3	−0.0406 (4)	0.2702 (4)	0.2478 (9)	0.0246 (12)	
C2	0.0686 (4)	0.2489 (4)	0.3410 (8)	0.0266 (14)	
H2	0.0914	0.1852	0.3698	0.032*	
C1	0.1440 (5)	0.3226 (4)	0.3914 (8)	0.0263 (13)	
C6	0.1099 (5)	0.4148 (4)	0.3463 (9)	0.0344 (16)	
H6	0.1616	0.4650	0.3786	0.041*	
C5	−0.0006 (5)	0.4342 (4)	0.2534 (10)	0.0384 (15)	
H5	−0.0242	0.4977	0.2241	0.046*	
C4	−0.0754 (5)	0.3611 (4)	0.2040 (10)	0.0337 (14)	
H4	−0.1505	0.3739	0.1401	0.040*	
C7	0.2650 (5)	0.3091 (4)	0.4934 (8)	0.0264 (13)	
C8	0.4049 (5)	0.1813 (4)	0.6035 (8)	0.0250 (13)	
C9	0.4207 (4)	0.0857 (4)	0.6545 (8)	0.0262 (13)	
C10	0.5238 (5)	0.0478 (4)	0.7497 (8)	0.0313 (14)	
H10	0.5320	−0.0174	0.7838	0.038*	
C11	0.6131 (5)	0.1073 (5)	0.7928 (9)	0.0346 (15)	
H11	0.6841	0.0831	0.8575	0.042*	
C12	0.6003 (5)	0.2006 (4)	0.7434 (8)	0.0339 (15)	
H12	0.6630	0.2406	0.7739	0.041*	
C13	0.4981 (4)	0.2388 (4)	0.6497 (8)	0.0298 (14)	
H13	0.4916	0.3041	0.6167	0.036*	
H1N	0.253 (6)	0.161 (5)	0.460 (10)	0.07 (3)*	

### Atomic displacement parameters (Å^2^)

**Table:**

	*U*^11^	*U*^22^	*U*^33^	*U*^12^	*U*^13^	*U*^23^
Cl1	0.0180 (6)	0.0357 (8)	0.0445 (8)	−0.0025 (6)	−0.0009 (8)	−0.0003 (8)
O1	0.023 (2)	0.030 (3)	0.064 (3)	−0.001 (2)	−0.001 (2)	−0.002 (2)
O2	0.021 (2)	0.035 (3)	0.061 (3)	−0.0019 (19)	−0.004 (2)	0.001 (2)
O3	0.034 (3)	0.028 (3)	0.072 (3)	−0.002 (2)	0.003 (2)	0.012 (2)
N1	0.017 (2)	0.028 (3)	0.032 (3)	−0.002 (2)	−0.002 (2)	0.000 (2)
N2	0.017 (3)	0.032 (3)	0.041 (3)	−0.002 (2)	0.006 (2)	−0.001 (2)
C3	0.018 (3)	0.028 (3)	0.028 (3)	−0.001 (2)	0.005 (3)	−0.002 (3)
C2	0.018 (3)	0.030 (3)	0.031 (3)	0.001 (3)	0.004 (2)	0.003 (2)
C1	0.019 (3)	0.031 (4)	0.028 (3)	0.000 (3)	0.005 (2)	0.003 (2)
C6	0.023 (3)	0.028 (4)	0.048 (4)	−0.001 (3)	0.002 (3)	−0.001 (3)
C5	0.024 (3)	0.033 (4)	0.053 (4)	0.007 (2)	0.000 (4)	0.009 (4)
C4	0.022 (3)	0.039 (4)	0.039 (3)	0.007 (3)	0.005 (3)	0.005 (3)
C7	0.022 (3)	0.027 (3)	0.027 (3)	−0.002 (3)	0.001 (2)	0.003 (3)
C8	0.016 (3)	0.031 (4)	0.025 (3)	0.001 (3)	0.001 (2)	0.000 (3)
C9	0.015 (3)	0.029 (3)	0.032 (3)	−0.004 (2)	0.003 (2)	−0.001 (3)
C10	0.023 (3)	0.029 (4)	0.040 (4)	0.001 (3)	0.005 (3)	0.003 (3)
C11	0.018 (3)	0.043 (4)	0.041 (4)	−0.002 (3)	0.003 (3)	−0.002 (3)
C12	0.017 (3)	0.042 (4)	0.040 (4)	−0.004 (3)	0.005 (3)	−0.004 (3)
C13	0.022 (3)	0.028 (3)	0.038 (3)	0.002 (3)	0.005 (3)	0.002 (3)

### Geometric parameters (Å, º)

**Table:**

Cl1—C3	1.735 (5)	C6—H6	0.9500
O1—C7	1.212 (6)	C5—C4	1.381 (7)
O2—N2	1.247 (5)	C5—H5	0.9500
O3—N2	1.219 (6)	C4—H4	0.9500
N1—C7	1.376 (7)	C8—C9	1.396 (7)
N1—C8	1.405 (7)	C8—C13	1.397 (7)
N1—H1N	0.98 (7)	C9—C10	1.397 (8)
N2—C9	1.474 (7)	C10—C11	1.374 (8)
C3—C4	1.365 (7)	C10—H10	0.9500
C3—C2	1.388 (7)	C11—C12	1.360 (8)
C2—C1	1.392 (7)	C11—H11	0.9500
C2—H2	0.9500	C12—C13	1.387 (7)
C1—C6	1.382 (7)	C12—H12	0.9500
C1—C7	1.513 (8)	C13—H13	0.9500
C6—C5	1.396 (7)		
			
C7—N1—C8	127.8 (5)	C5—C4—H4	120.2
C7—N1—H1N	126 (4)	O1—C7—N1	124.1 (5)
C8—N1—H1N	106 (4)	O1—C7—C1	121.3 (5)
O3—N2—O2	121.9 (5)	N1—C7—C1	114.6 (5)
O3—N2—C9	119.0 (5)	C9—C8—C13	116.8 (5)
O2—N2—C9	119.1 (5)	C9—C8—N1	120.8 (5)
C4—C3—C2	121.8 (5)	C13—C8—N1	122.4 (5)
C4—C3—Cl1	119.3 (4)	C8—C9—C10	122.7 (5)
C2—C3—Cl1	118.9 (4)	C8—C9—N2	122.5 (5)
C3—C2—C1	118.7 (5)	C10—C9—N2	114.8 (5)
C3—C2—H2	120.6	C11—C10—C9	118.3 (6)
C1—C2—H2	120.6	C11—C10—H10	120.9
C6—C1—C2	120.0 (5)	C9—C10—H10	120.9
C6—C1—C7	116.0 (5)	C12—C11—C10	120.3 (6)
C2—C1—C7	124.0 (5)	C12—C11—H11	119.8
C1—C6—C5	120.0 (6)	C10—C11—H11	119.8
C1—C6—H6	120.0	C11—C12—C13	121.7 (6)
C5—C6—H6	120.0	C11—C12—H12	119.2
C4—C5—C6	119.9 (6)	C13—C12—H12	119.2
C4—C5—H5	120.0	C12—C13—C8	120.1 (5)
C6—C5—H5	120.0	C12—C13—H13	119.9
C3—C4—C5	119.5 (5)	C8—C13—H13	119.9
C3—C4—H4	120.2		
			
O2—O2—N2—O3	0.0 (3)	C7—N1—C8—C13	−18.1 (9)
O2—O2—N2—C9	0.0 (6)	C13—C8—C9—C10	0.9 (9)
C4—C3—C2—C1	0.2 (9)	N1—C8—C9—C10	−179.8 (5)
Cl1—C3—C2—C1	−179.1 (4)	C13—C8—C9—N2	−179.1 (5)
C3—C2—C1—C6	−0.8 (8)	N1—C8—C9—N2	0.2 (8)
C3—C2—C1—C7	179.9 (5)	O3—N2—C9—C8	−166.2 (6)
C2—C1—C6—C5	1.1 (9)	O2—N2—C9—C8	15.1 (8)
C7—C1—C6—C5	−179.6 (5)	O2—N2—C9—C8	15.1 (8)
C1—C6—C5—C4	−0.9 (10)	O3—N2—C9—C10	13.8 (7)
C2—C3—C4—C5	0.0 (10)	O2—N2—C9—C10	−164.8 (5)
Cl1—C3—C4—C5	179.3 (5)	O2—N2—C9—C10	−164.8 (5)
C6—C5—C4—C3	0.3 (10)	C8—C9—C10—C11	−0.6 (9)
C8—N1—C7—O1	3.7 (10)	N2—C9—C10—C11	179.4 (5)
C8—N1—C7—C1	−176.5 (5)	C9—C10—C11—C12	0.0 (9)
C6—C1—C7—O1	9.1 (8)	C10—C11—C12—C13	0.2 (9)
C2—C1—C7—O1	−171.5 (6)	C11—C12—C13—C8	0.1 (9)
C6—C1—C7—N1	−170.6 (5)	C9—C8—C13—C12	−0.6 (8)
C2—C1—C7—N1	8.7 (8)	N1—C8—C13—C12	−179.9 (5)
C7—N1—C8—C9	162.7 (6)		

### Hydrogen-bond geometry (Å, º)

**Table:**

*D*—H···*A*	*D*—H	H···*A*	*D*···*A*	*D*—H···*A*
N1—H1*N*···O2	0.98 (7)	1.75 (7)	2.612 (6)	144 (6)
C10—H10···O1^i^	0.95	2.39	3.158 (7)	138

Symmetry code: (i) −*x*+1, *y*−1/2, −*z*+3/2.
