# Supplementary figures and images for: Crystal structure of 3-chloro-N-(2-nitro­phen­yl)benzamide
Source: Acta Crystallogr E Crystallogr Commun. 2015 Aug 22;71(Pt 9):o674. doi: 10.1107/S2056989015014620 (PMC4555397; doi:10.1107/S2056989015014620)

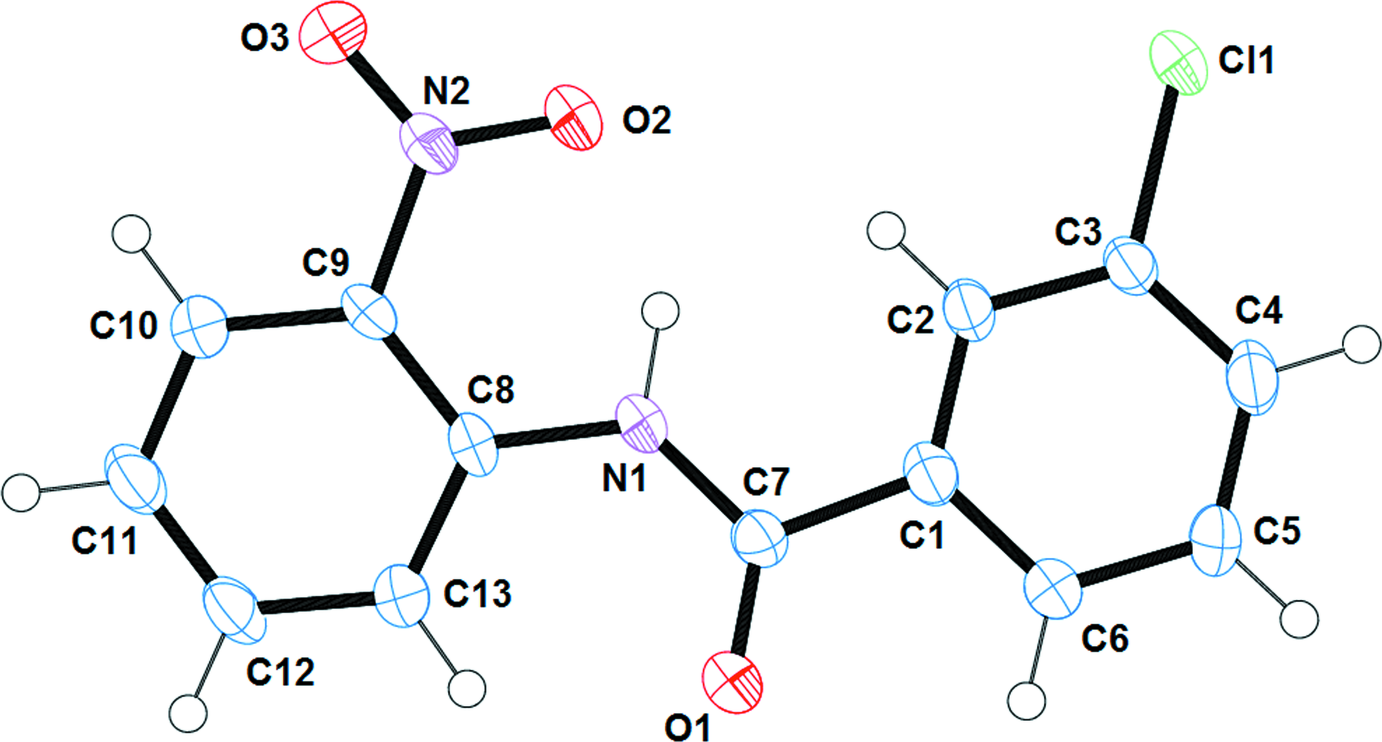

Supplement: Supplementary file 4 [file e-71-0o674-fig1.tif]

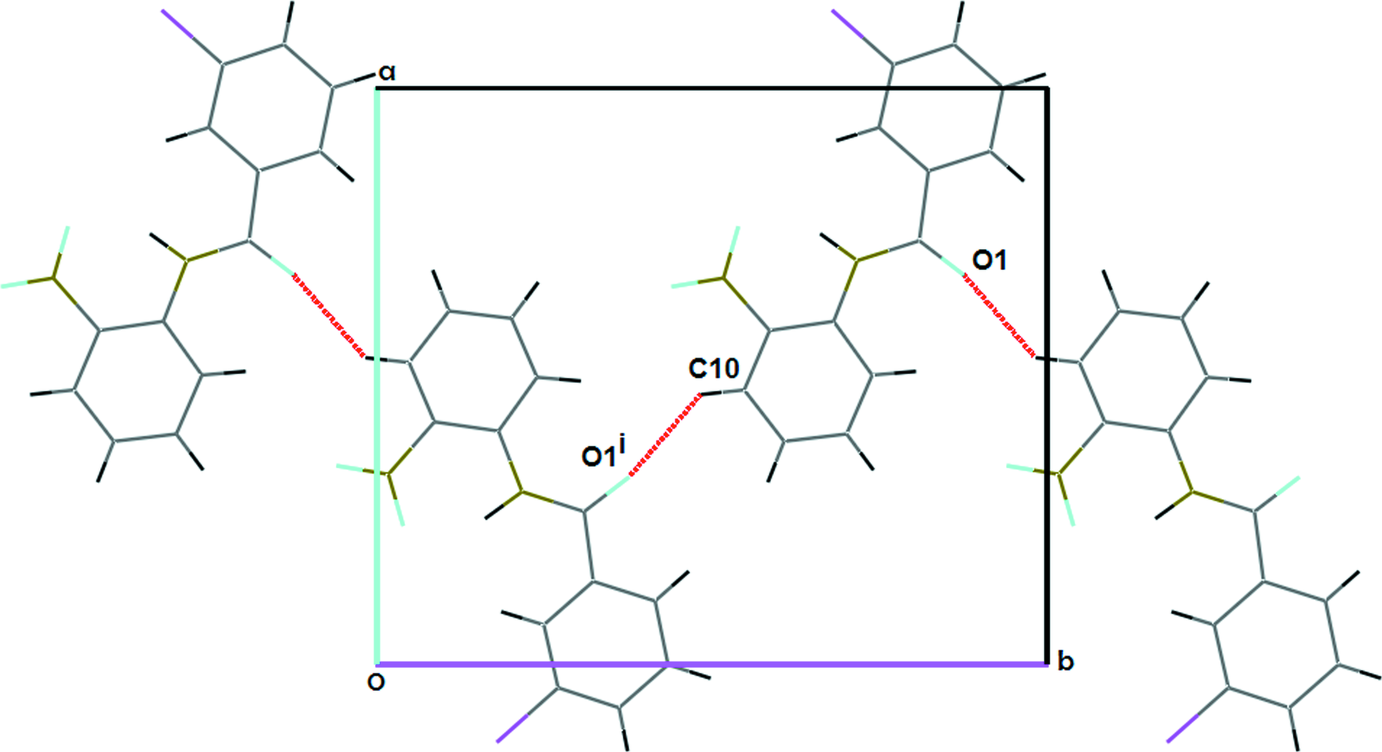

Supplement: Supplementary file 5 [file e-71-0o674-fig2.tif]
